# Post-Ripening and Key Glycosyltransferase Catalysis to Promote Sweet Mogrosides Accumulation of *Siraitia grosvenorii* Fruits

**DOI:** 10.3390/molecules28124697

**Published:** 2023-06-11

**Authors:** Shengrong Cui, Yimei Zang, Lei Xie, Changming Mo, Jiaxian Su, Xunli Jia, Zuliang Luo, Xiaojun Ma

**Affiliations:** 1 Institute of Medicinal Plant Development, Chinese Academy of Medical Sciences, Peking Union Medical College, Beijing 100193, Chinaleixie1996@163.com (L.X.);; 2 Biomedicine College, Beijing City University, Beijing 100094, China; 3 Guangxi Crop Genetic Improvement and Biotechnology Lab, Guangxi Academy of Agricultural Sciences, Nanning 530007, China

**Keywords:** *Siraitia grosvenorii*, mogrosides, metabolomics, post-ripening, UGT94-289-3

## Abstract

Sweet mogrosides are not only the primary bioactive ingredient in *Siraitia grosvenorii* fruits that exhibit anti-tussive properties and expectorate phlegm, but they are also responsible for the fruit’s sweetness. Increasing the content or proportion of sweet mogrosides in *Siraitia grosvenorii* fruits is significant for improving their quality and industrial production. Post-ripening is an essential step in the post-harvest processing of *Siraitia grosvenorii* fruits, but the underlying mechanism and condition of post-ripening on *Siraitia grosvenorii* quality improvement need to be studied systematically. Therefore, this study analyzed the mogroside metabolism in *Siraitia grosvenorii* fruits under different post-ripening conditions. We further examined the catalytic activity of glycosyltransferase UGT94-289-3 *in vitro*. The results showed that the post-ripening process of fruits could catalyze the glycosylation of bitter-tasting mogroside IIE and III to form sweet mogrosides containing four to six glucose units. After ripening at 35 °C for two weeks, the content of mogroside V changed significantly, with a maximum increase of 80%, while the increase in mogroside VI was over twice its initial amount. Furthermore, under the suitable catalytic condition, UGT94-289-3 could efficiently convert the mogrosides with less than three glucose units into structurally diverse sweet mogrosides, i.e., with mogroside III as the substrate, 95% of it can converted into sweet mogrosides. These findings suggest that controlling the temperature and related catalytic conditions may activate UGT94-289-3 and promote the accumulation of sweet mogrosides. This study provides an effective method for improving the quality of *Siraitia grosvenorii* fruits and the accumulation of sweet mogrosides, as well as a new economical, green, and efficient method for producing sweet mogrosides.

## 1. Introduction

*Siraitia grosvenorii*, also known as Luo han guo or monk fruit, is a unique Chinese medicine with both medicinal and edible values; it is listed in all editions of the Chinese Pharmacopoeia. This traditional Chinese medicine (TCM) has been found to have numerous health benefits, including cough relief and phlegm reduction, clearing heat, moisturizing the lungs, promoting vocalization, lubricating the intestines, and facilitating bowel movements [1,2,3]. It has also been reported to have hypoglycemic and hypolipidemic effects [4], anti-inflammatory and anti-asthmatic properties [5,6,7], antioxidant activity [8], anti-cancer effects [9], anti-fibrosis effects [10], modulate blood glucose levels and gut microbiota [11,12,13,14], and have immune-enhancing properties [15]. Mogrosides, which have been found to improve the human immune system, liver function, and blood glucose, are the main active ingredient responsible for the cough-relieving and expectorant properties of *Siraitia grosvenorii* fruits [16,17,18,19]. Mogrosides are also a type of high-sweet, low-calorie, safe and non-toxic, non-bitter aftertasting, stable, and freely soluble functional natural sweetener that is found all over the world [20,21]. In fact, simonoside I, mogroside IV, mogroside V, and isosigroside V have been shown to be 563-, 392-, 425-, and 500-fold sweeter than a 0.5% sucrose solution (*w*/*v*), respectively [22,23]. In China and Japan, mogroside extract has been approved as a healthy food sugar substitute for patients with obesity and diabetes [1,24]. It received GRAS certification from the US FDA in 2011 and successfully entered the US market [25]. It currently has market access in more than 20 countries, with over 5000 products available, indicating its broad market potential.

Mogrosides are cucurbitane glycosides with the aglycon mogrol in common. According to research, the number and location of glucose units are responsible for taste perception [26]. Mogroside IA, mogroside IIE, mogroside III, and related isomers are formed when the total number of glucose units linked at positions 3 and 24 of mogrol is between one and three. After linking four to six glucose units, sweet mogrosides with high sweetness, such as simonoside I, mogroside V, mogroside VI, and related isomers, are formed. Sweeteners are made from mogroside mixtures containing four to six glucose units [27,28]. Young fruits contain mostly bitter-tasting mogrosides during the early stages of fruit development but the main compound within 30 days after pollination (DAP) is mogroside IIE [29]. When the fruit ripens, the content of sweet mogrosides such as mogroside V rises sharply after 50 DAP and tends to stabilize around 80 DAP [30]. However, sweet mogrosides account for only 70% of the total mogrosides in ripe fruits, and approximately 30% of the bitter-tasting mogrosides, such as mogroside IIE and mogroside III, are discarded in the mogroside extraction industry. Furthermore, a significant number of fruits (containing bitter-tasting mogrosides) fail to mature in the late stage of fruit production due to weather and plant nutrient consumption. Understanding the pattern of increasing the content of sweet mogrosides in *Siraitia grosvenorii* fruits via post-ripening processing can thus provide guidance for the design of the post-ripening process. Additionally, if the bitter-tasting waste from the agricultural production and industrial extraction of *Siraitia grosvenorii* fruits, which contains a significant amount of mogroside IIE and mogrosides III, can be transformed into sweet mogrosides via a biotechnological method, it could potentially serve as a sweet mogroside production pathway as well as an effective waste utilization method.

Fruit ripening involves a series of physiological, biochemical, and organoleptic changes that finally leads to the development of a soft edible ripe fruit with desirable quality attributes. Carbohydrates play an important role in the fruit ripening process [31]. Typically, the accumulation of components such as fructose, sorbitol, glucose, and sucrose causes an increase in sweetness during fruit ripening. The sweet substances in *Siraitia grosvenorii* fruits, in particular, are mogrosides, which are triterpenoid secondary metabolites. The glycosyltransferase in the synthetic pathway is primarily responsible for the accumulation of sweet mogrosides. Currently, the synthetic pathway of mogrosides has been successfully elucidated. Through continuous glycosylation of the substrate mogroside IIE, the downstream glycosyltransferase UGT94-289-3 can form simonoside I, mogroside IV, mogroside V, and mogroside VI [32]. Glycosylation, catalyzed by UGT94-289-3, is a key modification reaction in the biosynthesis of sweet mogroside, and UGT94-289-3 determines the number and position of the sugar groups in mogroside. As a result, UGT94-289-3 catalytic activation will promote the accumulation of sweet mogroside.

In this study, 80-day-old fruits of *Siraitia grosvenorii* were used as primary materials. The changes in sweet mogrosides under different temperature conditions were analyzed using metabolomics. In addition, we cloned and heterologously expressed the UGT94-289-3 gene, purified the protein, and established the catalytic system of UGT94-289-3 *in vitro*. Following previous studies [32], monomeric mogroside III and mogroside extracts were utilized as substrates for *in vitro* catalysis. This study provides a scientific basis for optimizing the post-ripening processing technology of *Siraitia grosvenorii* fruits and offers a novel approach for producing sweet mogrosides.

## 2. Results

### 2.1. Mogrosides Metabolic Profile in Post-Ripening Samples

In this study, the Orbitrap Exploris 120 mass spectrometer was used to qualitatively analyze the mogrosides in the fruit samples subjected to post-ripening treatment at different temperatures. A total of 29 mogrosides were deduced by the accurate molecular weight of high-resolution mass spectra, fragment ions of MS2 spectra, and retention time. Comparison with the literature and reference standards further confirmed the identification of the mogrosides. Among these, 10 mogrosides were confirmed by comparing the retention time and the MS and MS2 spectra information with the reference standard; the results are presented in Table 1. In the negative ion full scan mode, the mogrosides mainly produced a relatively high-abundant adduct molecular ion peak [M+HCOOH−H]^−^ and a relatively low-abundant molecular ion peak [M−H]^−^. Meanwhile, the MS2 spectra mainly produced abundant fragment ions by the successive neutral losses of glucosyl moiety (162 Da). As an example, for mogroside V, an adduct molecular ion peak at *m*/*z* 1331.6472 for [M+HCOOH−H]^−^ and a molecular ion peak at *m*/*z* 1285.6421 for [M−H]^−^ were identified in full-scan mode, while the fragment ions at *m*/*z* 1123.5891, 961.5394, and 799.4803 were attributed to the sequential neutral loss of glucosyl moieties from the deprotonated ion at *m*/*z* 1285.6421 in the MS2 spectra. The mass spectra in the full-scan mode and targeted-MS2 mode are presented in Supplementary Figure S1.

**Figure 1:**
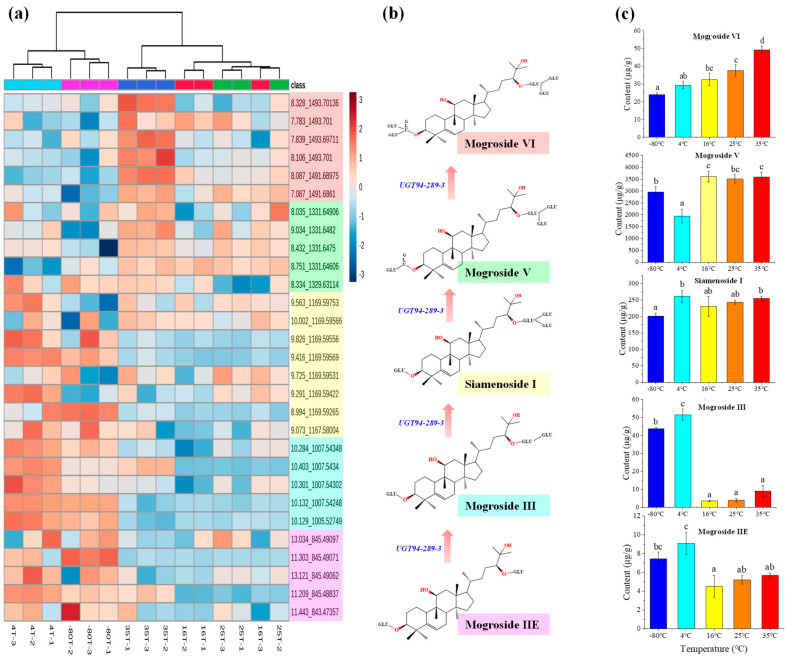
Analysis of mogroside metabolites in the post-ripening fruits of *Siraitia grosvenorii*. (**a**) Cluster heatmap of mogroside metabolites in post-ripening fruits, the metabolites under the pink background represent mogroside IIE and its isomers which contain two glucosyl units, the metabolites under the blue background represent mogroside III and its isomers which contain three glucosyl units, the metabolites under the yellow background represent siamenoside I and its isomers which contain four glucosyl units, the metabolites under the green background represent mogroside V and its isomers which contain five glucosyl units, and the metabolites under the yellow background represent mogroside VI and its isomers which contain six glucosyl units. (**b**) The synthesis pathway of sweet mogrosides, glycosyltransferase UGT94-289-3 can continuously catalyze glycosylation using mogroside IIE as a substrate to obtain various sweet mogrosides. (**c**) The quantitative results of five mogrosides (mogroside IIE, mogroside III, siamenoside I, mogroside V, and mogroside VI), the lowercase letters represent statistically significant differences (*p* < 0.5).

**Figure 2:**
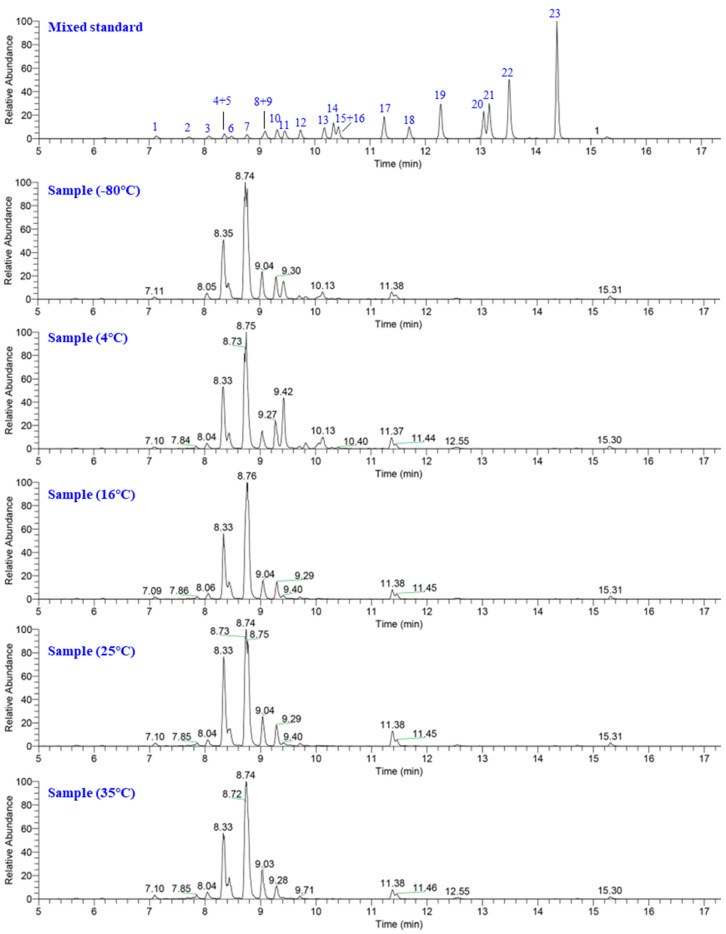
Extraction ion chromatography of the mixed standard and samples from different treatment groups. The peaks are as follows: 1: 11-oxo-morgroside VI, peak 2: mogroside VI, peak 3: 11-oxo-mogroside V, peak 4: mogroside VIB, peak 5: 11-epi-morgroside V, peak 6: mogroside VIA, peak 7: mogroside V, peak 8: isomogroside V, peak 9: 11-O-Siamenoside I, peak 10: siamenoside I, peak 11: mogroside IVA, peak 12: mogroside IV, peak 13: mogroside III, peak 14: mogroside IIIE, peak 15: mogroside IIIA2, peak 16: 11-deoxymorgroside V, peak 17: mogroside IIE, peak 18: mogroside IIIA1, peak 19: mogroside IIA2, peak 20: mogroside IIA1, peak 21: mogroside IIA, peak 22: mogroside IE, and peak 23: mogroside IA.

A cluster analysis was conducted using the relative contents of the identified metabolites in the samples from different treatment groups (Figure 1a). The clustering results revealed that a total of 15 samples from five treatment groups at −80 °C, 4 °C, 16 °C, 25 °C, and 35 °C were clustered into two subgroups. Subgroup I consisted of six samples from the −80 °C and 4 °C treatments, while subgroup II included nine samples from the 16 °C, 25 °C, and 35 °C treatments. Samples from the same treatment groups were all clustered into one subset except for the 16 °C treatment group. The clustering results demonstrated that the relative contents of most mogrosides were altered by different temperature treatments, and the difference between the samples within the treatment group was small. According to the classification of mogrosides, an increase in temperature can promote the accumulation of sweet mogrosides containing four to six glucose units and decrease the relative content of bitter-tasting mogrosides possessing fewer glucose units such as mogroside IIe and III. The extracted ion spectra of samples from the different treatment groups more clearly reflected the changes in mogrosides among the different treatments (Figure 2), and the variation pattern of mogrosides was consistent with that observed during fruit growth cycles. In the synthetic pathway of mogrosides, bitter-tasting mogrosides possessing fewer glucose units are constantly consumed as substrates and eventually formed sweet mogrosides (Figure 1b).

Moreover, eight mogrosides including mogroside VI, mogroside V, siamenoside I, mogroside IVa, mogroside IV, mogroside III, mogroside IIe, and mogroside IA were quantitatively analyzed by extracting the target ions 1493.69, 1331.64, 1169.59, 1007.54, 845.48, and 683.43, respectively, in the full scan spectra. Quantitation was performed using external standard calibration. The results are presented in Figure 1c and Table 2. The quantitative analysis revealed significant changes in the content of mogrosides, with mogroside V increasing by 80% and mogroside VI increasing more than twice under different post-ripening conditions. These results indicate that an appropriate post-ripening process could significantly increase the content of sweet mogrosides.

Furthermore, the quantitative method was assessed by evaluating linearity, accuracy, precision, and repeatability. Calibration curves for eight mogrosides were constructed using six independent standard solutions (with concentrations of 0.5, 1, 2, 5, 10, and 20 μg/mL for all target analytes) and exhibited good linearity with correlation coefficients (γ) higher than 0.99. The intra-day and inter-day precision (RSD values) for the investigated mogrosides were less than 8.76% and 7.01%, respectively, and the average recoveries of all investigated mogrosides ranged from 93.45% to 105.49%. The repeatability for the investigated mogrosides was less than 8.05% (RSD values); the results are presented in Supplementary Table S1. These results indicate that the quantitative method is accurate and reliable.

### 2.2. In Vitro Catalysis of UGT94-289-3

**Figure 3:**
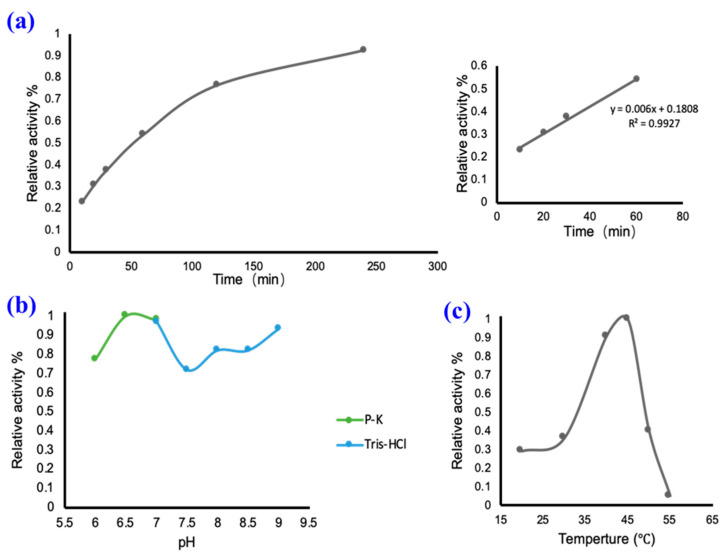
The effect of reaction conditions on the enzyme activity of UGT94-289-3. The enzyme activity was evaluated under different reaction times (**a**), pH values (**b**), and reaction temperatures (**c**).

The effects of glycosylation reaction conditions, including the reaction time, temperature, and pH, on the *in vitro* catalytic efficiency are presented in Figure 3. The catalytic activity increased linearly from 0 to 60 min, indicating a stable reaction efficiency during this time period. However, the slope gradually decreased after 60 min, indicating a reduced reaction efficiency. Two common buffers, PBS and Tris, were employed to examine the effect of pH on the enzymatic activity of UGT94-289-3. The catalytic efficiency remained above 70% within the pH range of 6–9, indicating a minimal impact of pH on enzyme activity. Notably, PBS at pH 6.5 yielded the highest catalytic efficiency. Conversely, the relative activity varied conspicuously within the temperature range of 20 to 55 °C. At room temperature (25 °C ± 5 °C), the relative activity of UGT94-289-3 was only about 30%, whereas 45 °C was found to be optimal. Furthermore, the enzyme activity decreased markedly with increasing temperature and disappeared almost entirely at 55 °C. By optimizing the catalytic conditions for UGT94-289-3, the best catalytic parameters for subsequent catalytic reactions were obtained, and efficient *in vitro* glycosylation reactions became possible.

**Figure 4:**
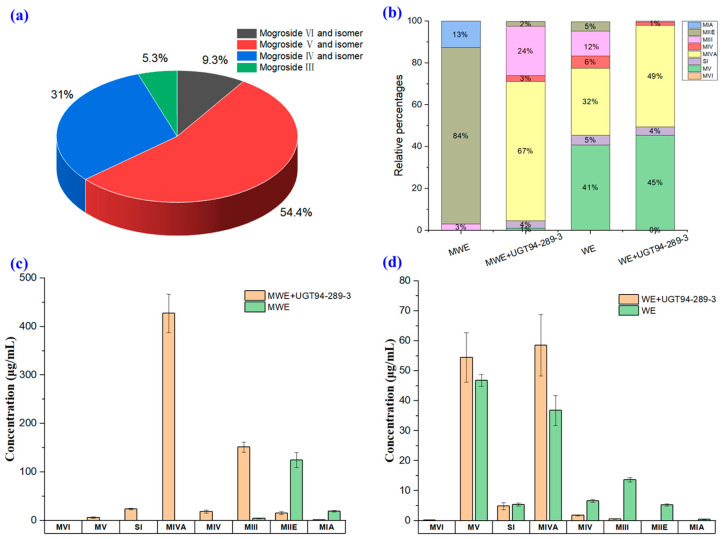
The catalytic results of mogroside III, MWE, and WE by UGT94-289-3. (**a**) The catalytic results of mogroside III as substrate; (**b**) the proportion change of eight mogrosides in extracts after catalysis; (**c**) the content of eight mogrosides in MWE and their catalytic products; and (**d**) the content of eight mogrosides in WE and their catalytic products.

In the optimized reaction system, mogroside III (0.5 mM) and extracts of WE and MWE were employed as the substrates for catalysis, and the extraction ion chromatography of mogroside metabolites is shown in Supplementary Figure S2. The results revealed the remarkable enzyme activity of UGT94-289-3, which converted mogroside III to a variety of sweet mogrosides with an impressive conversion yield of 95% (Figure 4a). Additionally, the composition of mogrosides in the catalytic products of extracts also changed significantly. A total of 52 mogrosides were identified using the metabolic identification methods from the catalytic products of the extracts, and their compound information is presented in Supplementary Table S2. A cluster analysis was conducted based on the relative content of compounds in different samples (Supplementary Figure S3) indicating that enzyme catalysis altered the relative content of mogrosides in the extracts and that the accumulation of sweet mogrosides increased. Mogroside III and mogroside I in the extract of MWE were largely converted to sweet mogrosides using this catalytic method, with the proportion of mogroside II decreasing from 84% to 2%, improving the production of mogroside IVA by 63%. The proportion of mogroside IVA in WE increased from 32% to 46% and mogroside V increased from 41% to 45% (Figure 4b). Overall, the proportion of sweet mogrosides increased significantly, while bitter-tasting mogrosides were consumed after catalysis. Moreover, eight mogrosides in the extract and their catalytic products were quantified by extracting target ions (Figure 4 and Supplementary Table S3). These results indicate that the current catalytic system is highly efficient at producing sweet mogrosides.

## 3. Materials and Methods

### 3.1. Chemicals, Reagents, and Plant Materials

Optima LC-MS grade methanol and acetonitrile were procured from Fisher Chemical (Fair Lawn, NJ, USA), while MS grade formic acid was purchased from Honeywell Fluka (Muskegon, MI, USA). Deionized water intended for HPLC-MS/MS analysis was purified using a Milli-Q system (Merck Millipore, Billerica, MA, USA). A total of 23 reference substances, namely mogroside VI, mogroside VIA, mogroside VIB, 11-oxo-morgroside VI, mogroside V, isomogroside V, 11-epi-morgroside V, 11-oxo-mogroside V, 11-deoxymorgroside V, siamenoside I, mogroside IVA, mogroside IV, 11-O-Siamenoside I, mogroside III, mogroside IIIA1, mogroside IIIA2, mogroside IIIE, mogroside IIE, mogroside IIA, mogroside IIA2, mogroside IIIA1, mogroside IA, and mogroside IE were purchased from Chengdu Must Bio-Technology Co., Ltd. (Chengdu, China).

*Siraitia grosvenorii* (Cultivar Qingpiguo) were grown at the Yongfu County cultivation base (Guilin, China, GPS coordinates are E110.030835 and N24.9637). The plant materials were authenticated by Prof Changming Mo from the Guangxi Academy of Agricultural Sciences, and voucher specimens were deposited at the Guangxi Crop Genetic Improvement and Biotechnology Lab of the Guangxi Academy of Agricultural Sciences (Nanning, China). About 80 green fruits (50 days after pollination) were collected for the extraction and isolation of mogrosides. In vitro, these extracts were used as substrates for enzyme catalysis. As post-ripening samples, approximately 60 ripe fruits (80 DAP) of uniform size were collected. On the day of collection, these post-ripening samples were transported to the laboratory and stored at predetermined temperatures (−80 °C, 4 °C, 16 °C, 25 °C, and 35 °C) for two weeks. Each treatment contained 12 whole fruit samples. Following the post-ripening experiment, the samples were evenly placed in an −80 °C freezer for metabolite analysis.

### 3.2. Instruments

The instruments used for this study included an ultrahigh-performance liquid chromatography (UPLC) system with a column oven and thermostated autosampler (Ultimate 3000, Thermo Fisher Scientific, Bremen, Germany); an Orbitrap Exploris 120 high-resolution mass spectrometer (Thermo Fisher Scientific, Bremen, Germany), equipped of a heated electrospray ionization source (HESI, Washington, DC, USA) and Xcalibur 4.5 data processing software; a KQ-400DE type ultrasonic cleaning machine (Kunshan Ultrasonic Instrument Co., Ltd., Kunshan, China); a Milli-Q purification system (Millipore, Bedford, MA, USA); a GZX-9070MBE type electric blast drying oven (Shanghai Boxun Industrial Co., LTD. Medical Equipment Factory, Shanghai, China); and a BT25S 1/100,000 electronic analytical balance and BS210S 1/10,000 electronic analytical balance (Sartorius Scientific Instrument (Beijing) Co., Ltd., Beijing, China).

### 3.3. Sample Preparation for Metabolic Analysis

To prepare the single standard stock solutions with a concentration of 1 mg/mL, an appropriate amount of 23 mogroside reference standards was accurately weighed and dissolved with methanol. A small amount of the single standard stock solution was mixed and diluted with methanol to obtain a mixed standard working solution with a concentration of 10 μg/mL. Fresh fruit samples were removed from the −80 °C refrigerator, and the pulp portion was crushed and ground into a fine powder with liquid nitrogen. Subsequently, 1.0 g of powder was precisely weighed and placed in a 50 mL centrifuge tube, and 20 mL of 80% methanol was added and vortexed. The supernatant was collected and filtered using a 0.22 M Millipore filter after an ultrasonic water-bath assisted extraction at room temperature at 40 kHz for 1 h.

The green fruits were dried in an oven and powdered. One kilogram of the powder was added to 10 times its volume of 95% ethanol (EtOH) and soaked for 24 h. The extraction was performed at reflux for 2 h and was repeated once. The solvent was then evaporated under reduced pressure to obtain an EtOH extract (65 g). Based on prior research [33], the extract was partitioned into an ethyl acetate (EtOAc)/H_2_O (1:1, v:v) mixture. The H_2_O phase was then extracted with n-butanol (n-BuOH), which yielded n-BuOH and H_2_O phases. Removal of the solvent under reduced pressure from the EtOAc, n-BuOH, and H_2_O phases yielded EtOAc, n-BuOH, and H_2_O fractions, respectively. The EtOAc fraction was further partitioned with n-hexane/MeOH/H_2_O (95:95:10, v:v:v), which yielded n-hexane and MeOH/H_2_O fractions. Removal of the solvent under reduced pressure from the n-hexane and MeOH/H_2_O phases yielded n-hexane and MeOH/H_2_O fractions, respectively. The extracts of the H_2_O (WE) and MeOH/H_2_O (MWE) fractions were dissolved in 80% methanol to prepare a 10 mg/mL extract solution, which was then used as the substrate for *in vitro* catalysis.

### 3.4. In Vitro Catalytic Experiment of UGT94-289-3

The gene encoding for UGT94-289-3 was amplified from the cDNA library of *S. grosvenorii* using the Gibson assembly method and cloned into the pET28a (+) plasmid (Merck Millipore, 69864). The recombinant plasmid was transformed into Rosetta (DE3) competent cells and grown in Lysogeny Broth medium at 37 °C with 220 rpm shaking until the optical density reached approximately 0.8. Induction was carried out with 0.5 mM IPTG, and the culture was further incubated at 18 °C with shaking at 180 rpm for an additional 12 h. The cells were harvested by centrifugation at 6000× *g* for 10 min at 4 °C, resuspended in lysis buffer (50 mM Tris-HCl, pH 8.0, 500 mM NaCl, 5% glycerol, and 10 mM imidazole), and lysed by sonication. The lysate was subsequently clarified by centrifugation at 18,000× *g* for 40 min at 4 °C, and loaded onto a pre-equilibrated Ni-NTA column (GE Life Sciences). The bound fraction was washed with 50 volumes of wash buffer (50 mM Tris 8.0, 500 mM NaCl, 100 mM imidazole), and then eluted with elution buffer (wash buffer supplemented with 200 mM imidazole).

To establish a stable and efficient catalytic system of UGT94-289-3 *in vitro*, we systematically optimized the reaction time, reaction temperature, and pH using mogroside III as the reaction substrate. Ultimately, *in vitro* glycosylation was performed using a reaction buffer consisting of 50 mM PBS (pH 6.5) and 5 mM β-mercaptoethanol. Mogroside III (0.5 mM) and the extracts of MWE and WE were used as substrates. The reaction was initiated by mixing 5 µL substrate, 8 mM UPG, and 10 µg of purified enzyme, to a final volume of 100 µL. The mixture was incubated at 45 °C for 12 h and then terminated by adding 50 µL of methanol. The glycosylated products were analyzed using the LC-MS metabolic analysis method.

### 3.5. Metabolic Analysis Based on LC-MS

The LC-MS system used for metabolic profiling acquisition consisted of a UPLC system and an Orbitrap Exploris 120 mass spectrometer. Separation was performed on a Waters ACQUITY UPLC HSS T3 column (2.1 mm × 100 mm, 1.7 µm). The mobile phase was comprised of 0.1% formic acid in water (A) and acetonitrile (B). The gradient program was as follows: 0 min, 5% B; 2.5 min, 20% B; 5 min, 23% B; 12 min, 40% B; 15–17 min, 100% B; and 17.1–20 min, 5% B. The flow rate was maintained at 0.3 mL/min during the entire chromatographic analysis process. The injection volume was 2 µL and the column temperature was set at 35 °C.

The Orbitrap Exploris 120 mass spectrometer was utilized for its ability to acquire MS/MS spectra on its data-dependent acquisition (DDA) mode, which was controlled by the acquisition software (Xcalibur, Thermo, Waltham, MA, USA). The heated electrospray ionization (HESI) was operated in negative mode with a spray voltage of 2.2 kV. The source parameters and MS scan parameters were set as follows: the capillary temperature and vaporizer temperature were set at 330 °C and 280 °C, respectively; the sheath and auxiliary gas were set at 35 and 15 Arb, respectively. The HRMS was acquired under full MS mode (resolution 60,000 FWHM) over the mass range *m*/*z* of 150–1500, with dd-MS2 resolution set at 15,000 FWHM and the collision energy set at 30/50/70 in the NCE mode. The full MS/dd-MS2 (full-scan and data-dependent MS/MS mode) was utilized for compound identification and the full MS scan was used for quantitation.

### 3.6. Statistical Analyses

For quantitative data, a one-way ANOVA test and the Student’s *t*-test were performed using the statistical analysis software Origin 8.0 version. The data are presented as the mean + SD and *p* < 0.05 was considered significant. To generate the heatmap, the online analysis platform MetaboAnalyst 5.0 (https://www.metaboanalyst.ca, accessed on 7 May 2023) was utilized.

## 4. Discussion

The analysis of the mogroside metabolic changes in fruit samples after post-ripening treatment revealed that the content of sweet mogrosides could be significantly increased with a suitable post-ripening temperature. Mogrosides containing more than four glucosyl units increased significantly at a temperature between 16 and 35 °C, while the content of mogrosides with less than three glucosyl groups decreased significantly. These results suggest that the temperature of post-ripening has a crucial effect on further glycosylation to form sweet mogrosides, and indicate that the highest proportion of sweet glycosides occurred at 35 °C. The greatest content of mogroside II was found in young fruits (0–30 DAP) during the fruit growth period (0–85 DAP), which were continuously glycosylated under the action of the UGT94-289-3. Sweet mogroside V started to form around 50 DAP and accumulated consistently, with the highest content being detected at the ripening stage (75–85 DAP). Meanwhile, the substantial consumption of mogrosides occurred [30,32]. We speculate that UGT94-289-3 plays an important role during post-ripening and that an appropriate temperature range can maximize its activity.

Harvest time, post-ripening, and drying temperature all have an impact on the quality and content of sweet mogrosides in *Siraitia grosvenorii* fruits during the harvesting and processing process. Post-ripening for saccharification is an important link in the post-harvest processing of *Siraitia grosvenorii* fruits, according to the traditional processing method. According to relevant studies, the sweet mogroside metabolites of *Siraitia grosvenorii* fruits changed significantly after seven days of storage at room temperature [34]. The content of sweet mogrosides such as mogroside V and mogroside VI increased, while the content of mogroside III and IV decreased. Generally, after 70 DAP the fruits begin to mature, and the content of mogroside V increases. However, the differences in fruit shape and appearance are too small to distinguish their quality during the ripening stage (75–85 DAP). Thus, if the harvesting period is incorrect, the quality of fruits will be seriously affected. Therefore, post-ripening for saccharification is critical for improving fruit quality. Furthermore, previous research has shown that environmental and genetic factors have a significant impact on the accumulation of sweet mogrosides [35,36]. Previously, we discovered that the content of mogroside V varied greatly between cultivars, ranging from 4.86 to 13.49 mg/g. To summarize, the variety selected, cultivation environment, harvest time, and post-harvest processing of *Siraitia grosvenorii* all affect the quality and yield of sweet mogrosides, so standardizing *Siraitia grosvenorii* production is critical. Although the accumulation of sweet glycosides has been partially understood, research on the physiological changes and molecular mechanisms of fruits during post-ripening is still lacking and warrants further investigation.

During our mogroside catalysis experiments, we made a crucial observation pertaining to the activity of UGT94-289-3. Specifically, we found that temperature had a significant impact on the activity of UGT94-289-3, with its relative activity being only 30% at a temperature below 30 °C and almost no activity being observed at temperatures above 55 °C. These findings offer valuable insights for the optimal temperature setting during the post-ripening process as well as for selecting suitable catalytic temperatures *in vitro*. After investigating the catalytic conditions of UGT94-289-3, we managed to establish a highly efficient and remarkably stable *in vitro* catalytic system that could serve as a reference for biotechnological or enzymatic engineering for the production of sweet mogrosides. When investigating the catalytic efficiency of this modified catalytic system using monomer mogroside III as substrate, the results were positive, with the substrate conversion exceeding 95%. However, we noted that a considerable proportion of bitter-tasting mogrosides remained unglycosylated in the extracts. Therefore, more work is needed to optimize different catalytic condition parameters and enhance catalytic efficiency further. With regard to catalytic products, more abundant catalytic products could be obtained *in vitro* than in the monk fruit.

Synthetic biology [37] and metabolic engineering [38] are currently being developed to provide a sustainable production approach for obtaining natural plant products, and related research has provided alternative pathways for the production of mogrosides. The research on the biosynthesis of mogrosides began in 2011 when Tang Qi et al. [30] obtained all the key enzyme genes involved in the biosynthesis pathway of mogrosides through transcriptome sequencing. Following that, Qiao et al. [39] created a yeast expression vector for the cucurbitadienol synthase gene and transformed it into yeast strains to produce a cucurbitadienol-producing engineered strain, successfully opening up a new pathway for the fermentation synthesis of mogroside in yeast. Furthermore, Itkin et al. [32] screened multiple mogroside V synthase genes and achieved the biosynthesis of mogrol (the aglycon of mogrosides) in *Saccharomyces cerevisiae*. They also discovered the key downstream glycosyltransferase enzymes, UGT720-269-1 and UGT94-289-3, responsible for the down-stream glycosylation which forms mogroside V. Li et al. [40] recently modified the UGTs (UDP-glucuronosyltransferases) involved in mogroside V synthesis, resulting in three UGT mutant enzymes that efficiently transformed the substrate mogroside IIE or IIIA into mogroside IV and V. These advancements in research have infused new life into the study of mogroside biosynthesis. However, the de novo synthesis of mogroside V in microbial chassis has not yet made a breakthrough.

Plant chassis cells have natural advantages over microbial chassis cells, providing an alternative for the heterologous synthesis of mogroside V. Based on the multi-gene assembly strategy of In-Fusion technology and 2A polypeptide binding, our research group successfully constructed a multi-gene expression vector carrying six key enzyme genes for mogroside V synthesis and transferred them into cucumber and tomato plants via agrobacterium conversion [41]. For the first time, cucumber plants containing the target product of mogroside V (about 587.0 ng/g DW) were obtained. Despite the fact that this study achieved the heterologous synthesis of mogroside V in cucumber fruit, its content was significantly lower than in *Siraitia grosvenorii*. Furthermore, plant heterologous synthesis using transgenic technology raises a number of food safety concerns, and the resulting mogrosides products cannot be considered natural sweeteners.

Enzyme engineering methods are currently widely used to produce mogrosides. On the one hand, most studies use enzyme catalysis to hydrolyze mogroside V into mogrosides with fewer glucosyl groups. For example, cellulase, α-glucosidase, and β-glucosidase were used to convert mogroside V into mogroside III, mogroside IIIE, and mogroside IIE [42,43]. In recent years, a large number of studies have been conducted to synthesize semenside I, mogroside V, VI, and other mogrosides by modifying the activity of glycotransferase and enzyme engineering [40,44]. In this study, the natural glycosyltransferase from *Siraitia grosvenorii* was used directly for catalysis, and by controlling the catalytic conditions, bitter glycosides could be easily, economically, and efficiently converted into various known sweet mogrosides in an environmentally friendly way. This spectrum of catalytic products could avoid the generation of new harmful by-products greatly.

This study has provided preliminary evidence to suggest that UGT94-289-3 plays a critical role in the post-ripening process of fruits. By optimizing the conditions and parameters of the post-ripening process, UGT94-289-3 can be activated to increase the content and proportion of sweet mogrosides. Moreover, *in vitro* catalysis can facilitate the rational utilization of waste resources in the monk fruit industry. For instance, the catalytic conversion of bitter-tasting mogroside IIE and III to produce sweet mogrosides is a potential production pathway and an effective method for waste utilization in agricultural production and the industrial extraction of *Siraitia grosvenorii*. Furthermore, since the fruit size of *Siraitia grosvenorii* is fixed after 30 DAP, and studies have shown that the content of total mogrosides after 50 DAP is comparable to that of the ripening stage, the early harvesting of green fruits followed by enzymatic catalysis can be used to yield the desired sweet mogrosides, greatly reducing the fruit growth cycle and increasing fruit yield. However, the results and conclusions we obtained are limited to post-ripening and *in vitro* catalysis experiments on small samples. Further verification is required by conducting larger-scale studies with an expanded sample size.

## 5. Conclusions

We identified the change regularity of mogrosides under different post-ripening temperature conditions using metabolomics analysis and confirmed the influence of post-ripening processing on the quality of *Siraitia grosvenorii* fruits. Furthermore, we developed and optimized a highly efficient and stable UGT94-289-3 *in vitro* catalytic system. The established catalytic system can easily, economically, and efficiently convert the bitter mogroside IIE and mogroside III into diversified sweet mogrosides in an environmentally friendly way. This research demonstrates an effective method for improving the quality of *Siraitia grosvenorii* fruits and the accumulation of sweet mogrosides, as well as a new economical and efficient method for producing sweet mogrosides. Simultaneously, it can realize the recycling and utilization of waste resources from the *Siraitia grosvenorii* industry, which has significant practical implications.

## Figures and Tables

**Table 1:** The list of identified mogroside metabolites in the post-ripening of *Siraitia grosvenorii* fruits.

Compound ID	Compound Name	Retention Time (min)	Molecular Formula	Calc. MW	MS (*m*/*z*)	MS2 Fragment (*m*/*z*)
8.328_1493.70136	Mogroside VIA	8.328	C_66_H_112_O_34_	1448.7035	1447.6924; 1493.7002	1447.6924; 1285.6514; 1123.5892; 961.5258; 799.4753; 637.4352
7.783_1493.701	Isomer of mogroside VI	7.783	C_66_H_112_O_34_	1448.7035	1447.6924; 1493.7002	1447.6924; 1285.6514; 1123.5892; 961.5258; 799.4753; 637.4352
7.839_1493.69711	Mogroside VI	7.839	C_66_H_112_O_34_	1448.7035	1447.6924; 1493.7002	1447.6924; 1285.6514; 1123.5892; 961.5258; 799.4753; 637.4352
8.106_1493.701	Isomer of mogroside VI	8.106	C_66_H_112_O_34_	1448.7035	1447.6924; 1493.7002	1447.6924; 1285.6514; 1123.5892; 961.5258; 799.4753; 637.4352
8.087_1491.68975	11-oxo-mogroside VI	8.087	C_66_H_110_O_34_	1446.6878	1447.6924; 1493.7002	1447.6924; 1285.6514; 1123.5892; 961.5258; 799.4753; 637.4352
7.087_1491.6861	Isomer of 11-oxo-mogroside VI	7.087	C_66_H_110_O_34_	1446.6878	1491.6869; 1445.6812	1445.6812; 1283.6067; 1121.5883; 797.4802
8.035_1331.64906	11-epi-morgroside V	8.035	C_60_H_102_O_29_	1286.6506	1331.6470; 1285.6514	1285.6514; 1123.5892; 961.5258; 799.4753; 637.4352
9.034_1331.6482	Isomogroside V	9.034	C_60_H_102_O_29_	1286.6506	1331.6470; 1285.6514	1285.6514; 1123.5892; 961.5258; 799.4753; 637.4352
8.432_1331.6475	Isomer of mogroside V	8.432	C_60_H_102_O_29_	1286.6506	1331.6470; 1285.6514	1285.6514; 1123.5892; 961.5258; 799.4753; 637.4352
8.751_1331.64606	Mogroside V	8.751	C_60_H_102_O_29_	1286.6506	1331.6470; 1285.6514	1285.6514; 1123.5892; 961.5258; 799.4753; 637.4352
8.334_1329.63114	11-oxo-mogroside V	8.334	C_60_H_100_O_29_	1284.635	1329.6327; 1283.6267	1283.6267; 1121.5709; 959.5239; 797.4661; 635.4319
9.563_1169.59753	Isomer of mogroside IV	9.563	C_54_H_92_O_24_	1124.5978	1169.5940; 1123.5892	1123.5892; 961.5366; 799.4835; 637.4275
10.002_1169.59566	Isomer of mogroside IV	10.002	C_54_H_92_O_24_	1124.5978	1169.5940; 1123.5892	1123.5892; 961.5366; 799.4835; 637.4275
9.826_1169.59556	Isomer of mogroside IV	9.826	C_54_H_92_O_24_	1124.5978	1169.5940; 1123.5892	1123.5892; 961.5366; 799.4835; 637.4275
9.416_1169.59569	Mogroside IVA	9.416	C_54_H_92_O_24_	1124.5978	1169.5940; 1123.5892	1123.5892; 961.5366; 799.4835; 637.4275
9.725_1169.59531	Mogroside IV	9.725	C_54_H_92_O_24_	1124.5978	1169.5940; 1123.5892	1123.5892; 961.5366; 799.4835; 637.4275
9.291_1169.59422	Siamenoside I	9.291	C_54_H_92_O_24_	1124.5978	1169.5940; 1123.5892	1123.5892; 961.5366; 799.4835; 637.4275
8.994_1169.59265	Isomer of mogroside IV	8.994	C_54_H_92_O_24_	1124.5978	1169.5940; 1123.5892	1123.5892; 961.5366; 799.4835; 637.4275
9.073_1167.58004	11-O-SiamenosideⅠ	9.073	C_54_H_90_O_24_	1122.5822	1167.5787; 1121.5725	1121.5725; 959.5148; 797.3799; 635.4319
10.284_1007.54348	Mogroside IIIE	10.284	C_48_H_82_O_19_	962.545	1007.5423; 961.5368	961.5368; 799.4839; 637.4294; 475.3731
10.403_1007.5434	Isomer of mogroside III	10.403	C_48_H_82_O_19_	962.545	1007.5423; 961.5368	961.5368; 799.4839; 637.4294; 475.3731
10.301_1007.54302	Mogroside IIIA2	10.301	C_48_H_82_O_19_	962.545	1007.5423; 961.5368	961.5368; 799.4839; 637.4294; 475.3731
10.132_1007.54248	Mogroside III	10.132	C_48_H_82_O_19_	962.545	1007.5423; 961.5368	961.5368; 799.4839; 637.4294; 475.3731
10.129_1005.52749	11-oxo-mogroside III	10.129	C_48_H_80_O_19_	960.5294	1005.5281; 959.5336	959.5336; 797.4736; 635.4147; 161.0452
13.034_845.49097	Mogroside IIa	13.034	C_42_H_72_O_14_	800.4922	845.4894; 799.4837	799.4837; 637.4323; 161.0454
11.303_845.49071	Isomer of mogroside IIE	11.303	C_42_H_72_O_14_	800.4922	845.4894; 799.4837	799.4837; 637.4323; 161.0454
13.121_845.49062	Mogroside IIA1	13.121	C_42_H_72_O_14_	800.4922	845.4894; 799.4837	799.4837; 637.4323; 161.0454
11.209_845.48837	mogroside IIE	11.209	C_42_H_72_O_14_	800.4922	845.4894; 799.4837	799.4837; 637.4323; 161.0454
11.443_843.47357	11-oxo-mogroside II	11.443	C_42_H_70_O_14_	798.4765	843.4745; 797.4689	797.4689; 635.4147 + B1:G30; 161.0454

**Table 2:** The quantitative results of the eight mogrosides in post-ripening fruits of *Siraitia grosvenorii* (mean value ± SD, *n* = 3).

Post-Ripening Temperature	Content of Mogrosides (μg/g)							
	Mogroside Ⅵ	Mogroside Ⅴ	Miamenoside Ⅰ	Mogroside ⅣA	Mogroside Ⅳ	Mogroside Ⅲ	Mogroside ⅡE	Mogroside IA
−80 °C	24.21 ± 2.45	2959.34 ± 222.72	200.99 ± 8.72	158.84 ± 9.98	23.08 ± 3.7	43.64 ± 0.75	7.44 ± 0.71	ND
4 °C	29.42 ± 2.11	1940.63 ± 296.75	261.41 ± 17.95	570.51 ± 59.09	42.58 ± 5.36	51.58 ± 3.21	9.09 ± 1.16	ND
16 °C	32.56 ± 3.48	3606.5 ± 219.61	230.52 ± 30.05	135.66 ± 32.65	30.79 ± 4.97	3.48 ± 0.37	4.53 ± 1.17	ND
25 °C	37.64 ± 3.25	3513.02 ± 175.61	243.55 ± 6.18	92.46 ± 25.95	42.99 ± 4.23	3.89 ± 0.94	5.21 ± 0.43	ND
35 °C	49.3 ± 2.1	3593.92 ± 191.57	255.52 ± 5.92	73.05 ± 11.45	71.03 ± 16.46	8.99 ± 3.18	5.68 ± 0.26	ND

Note: ND means not detected or below LOQ.

## Data Availability

Not applicable.

## Supplementary Materials

The following supporting information can be downloaded at: https://www.mdpi.com/article/10.3390/molecules28124697/s1, Figure S1: The mass spectra in full-scan mode and targeted-MS2 mode for mogrosides with different glucose units; Figure S2: The extraction ion chromatography of the catalytic products of mogroside III, MWE, and WE; Figure S3: The cluster heatmap of mogroside metabolites in the extracts and their catalytic products; Table S1: The results of the quantitative method validation; Table S2: The list of identified mogroside metabolites in the extracts and their catalytic products; Table S3: The quantitative results of eight mogrosides in extracts and their catalytic products.
