# Supplementary material for: Post-Ripening and Key Glycosyltransferase Catalysis to Promote Sweet Mogrosides Accumulation of Siraitia grosvenorii Fruits
Source: Molecules. 2023 Jun 11;28(12):4697. doi: 10.3390/molecules28124697 (PMC10303746; doi:10.3390/molecules28124697)
Supplement: Supplementary file 1 [file molecules-28-04697-s001.zip › molecules-2430252-supplementary.pdf]

(a)

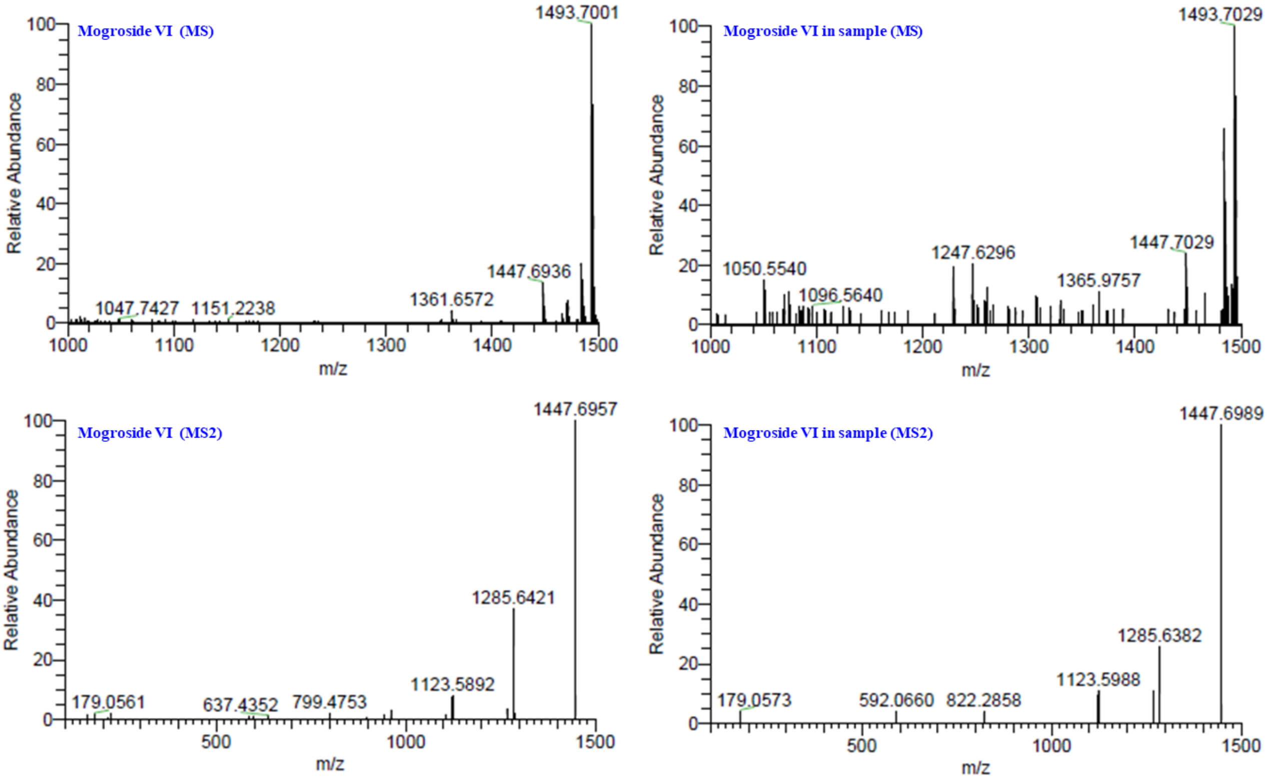
Mogroside VI (MS)
1493.7001
1447.6936
1361.6572
1047.7427
1151.2238
Mogroside VI in sample (MS)
1493.7029
1447.7029
1247.6296
1365.9757
1050.5540
1096.5640
Mogroside VI (MS2)
1447.6957
1285.6421
1123.5892
179.0561
637.4352
799.4753
Mogroside VI in sample (MS2)
1447.6989
1285.6382
1123.5988
179.0573
592.0660
822.2858
Relative Abundance
m/z

(b)

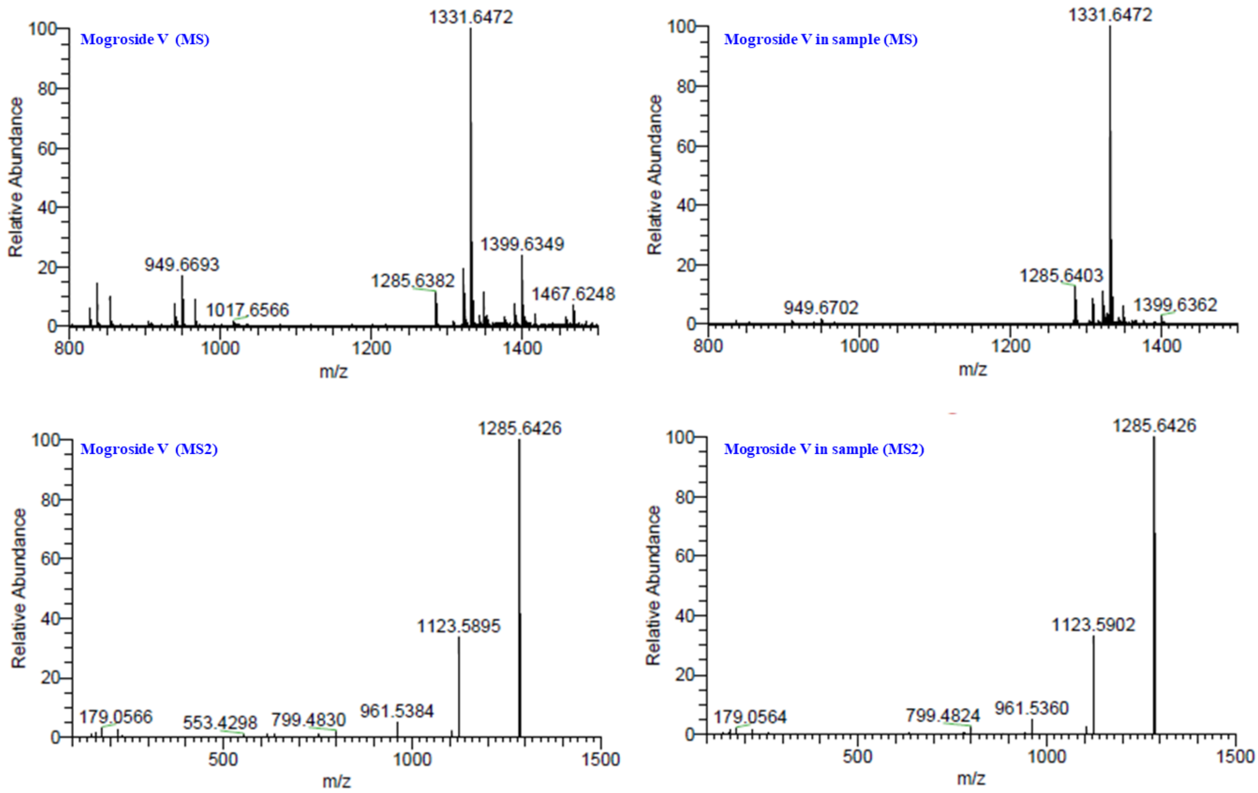
Mogroside V (MS)
1331.6472
1399.6349
949.6693
1285.6382
1467.6248
1017.6566
Mogroside V in sample (MS)
1331.6472
1285.6403
949.6702
1399.6362
Mogroside V (MS2)
1285.6426
1123.5895
179.0566
553.4298
799.4830
961.5384
Mogroside V in sample (MS2)
1285.6426
1123.5902
179.0564
799.4824
961.5360
Relative Abundance
m/z

(c)

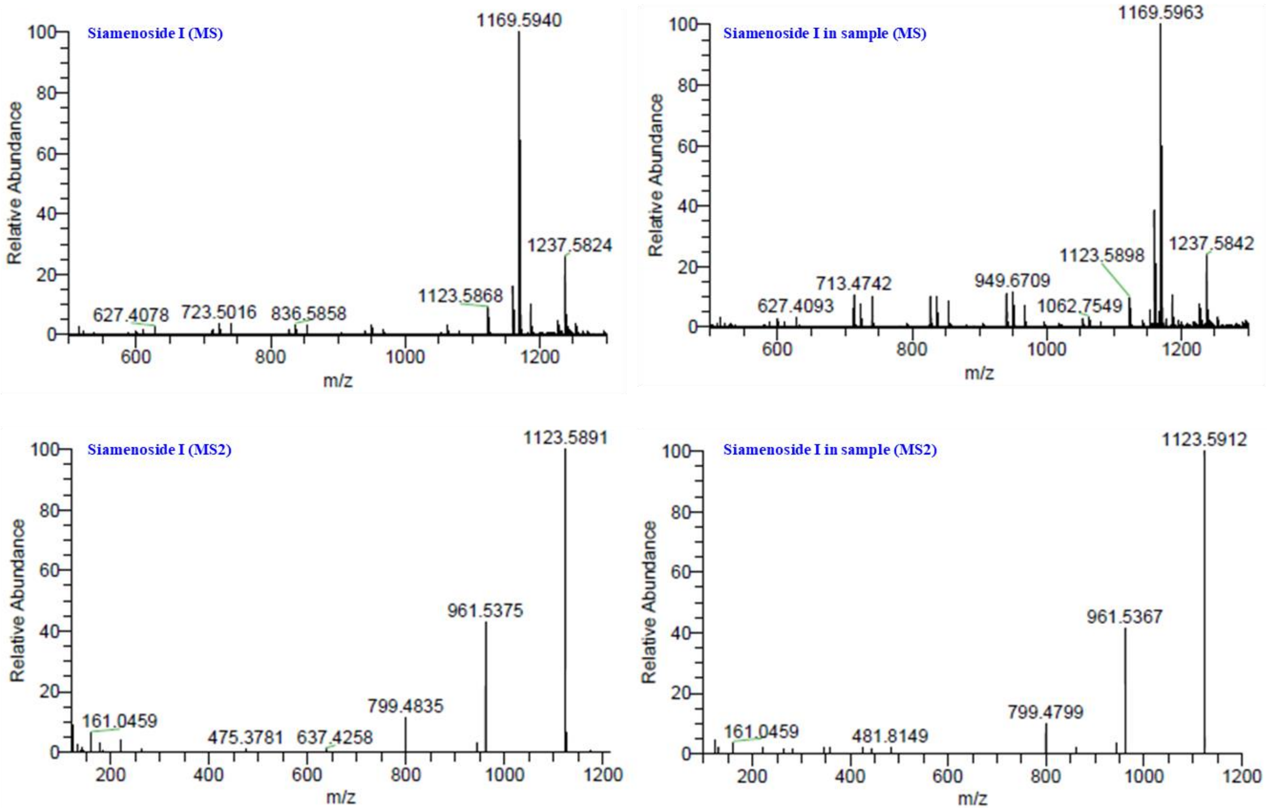

Siamenoside I (MS)
1169.5940
1237.5824
1123.5868
627.4078
723.5016
836.5858
Relative Abundance
m/z
Siamenoside I in sample (MS)
1169.5963
1123.5898
1237.5842
713.4742
949.6709
627.4093
1062.7549
Siamenoside I (MS2)
1123.5891
961.5375
799.4835
161.0459
475.3781
637.4258
Siamenoside I in sample (MS2)
1123.5912
961.5367
799.4799
161.0459
481.8149

(d)

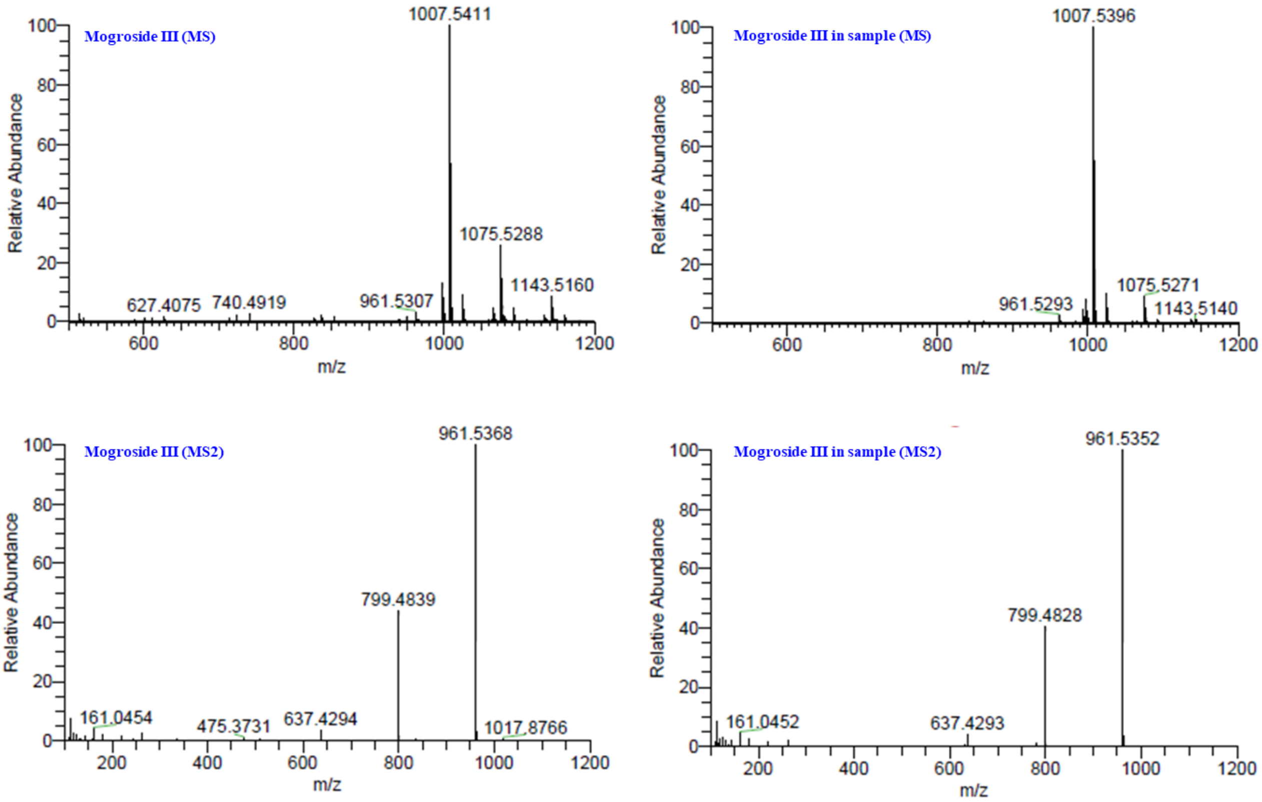

Mogroside III (MS)
1007.5411
1075.5288
1143.5160
627.4075
740.4919
961.5307
Relative Abundance
m/z
Mogroside III in sample (MS)
1007.5396
1075.5271
961.5293
1143.5140
Mogroside III (MS2)
961.5368
799.4839
161.0454
475.3731
637.4294
1017.8766
Mogroside III in sample (MS2)
961.5352
799.4828
161.0452
637.4293

(e)

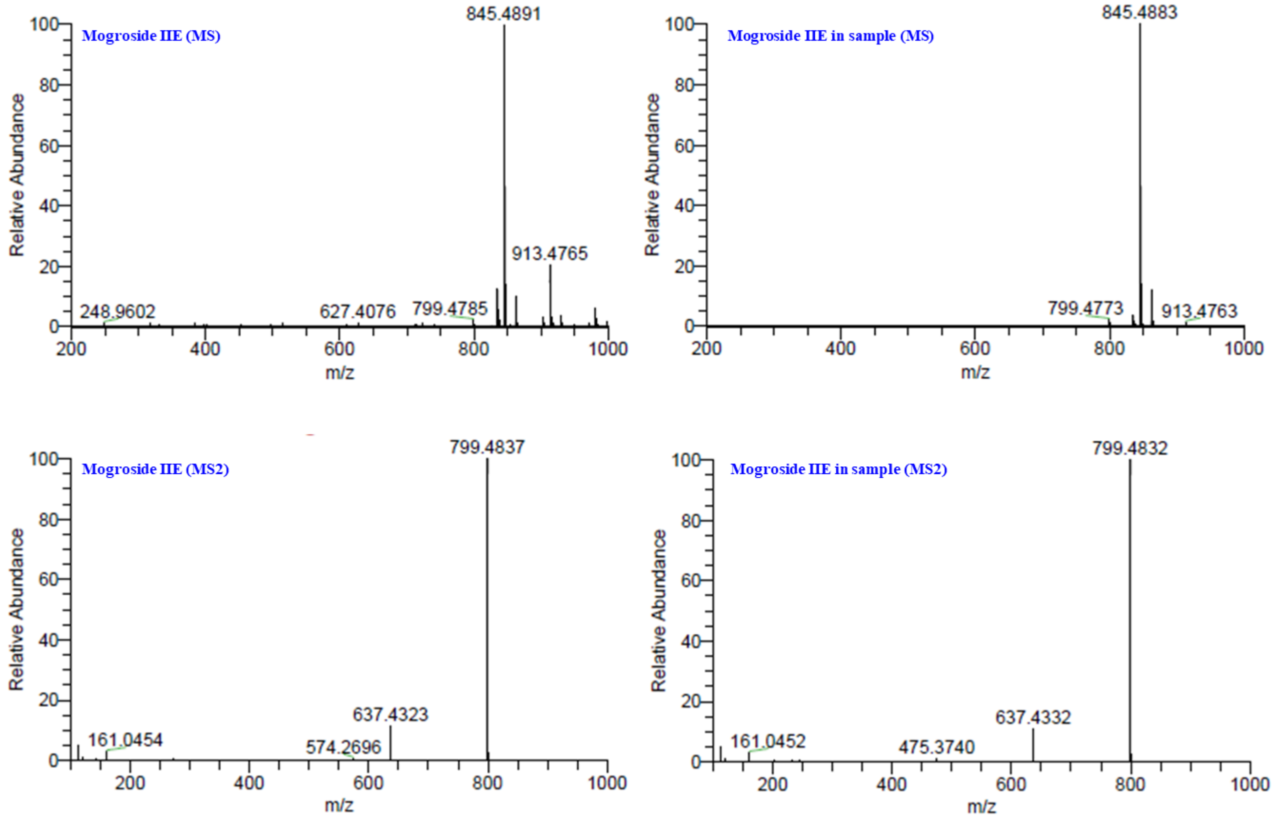

(f)

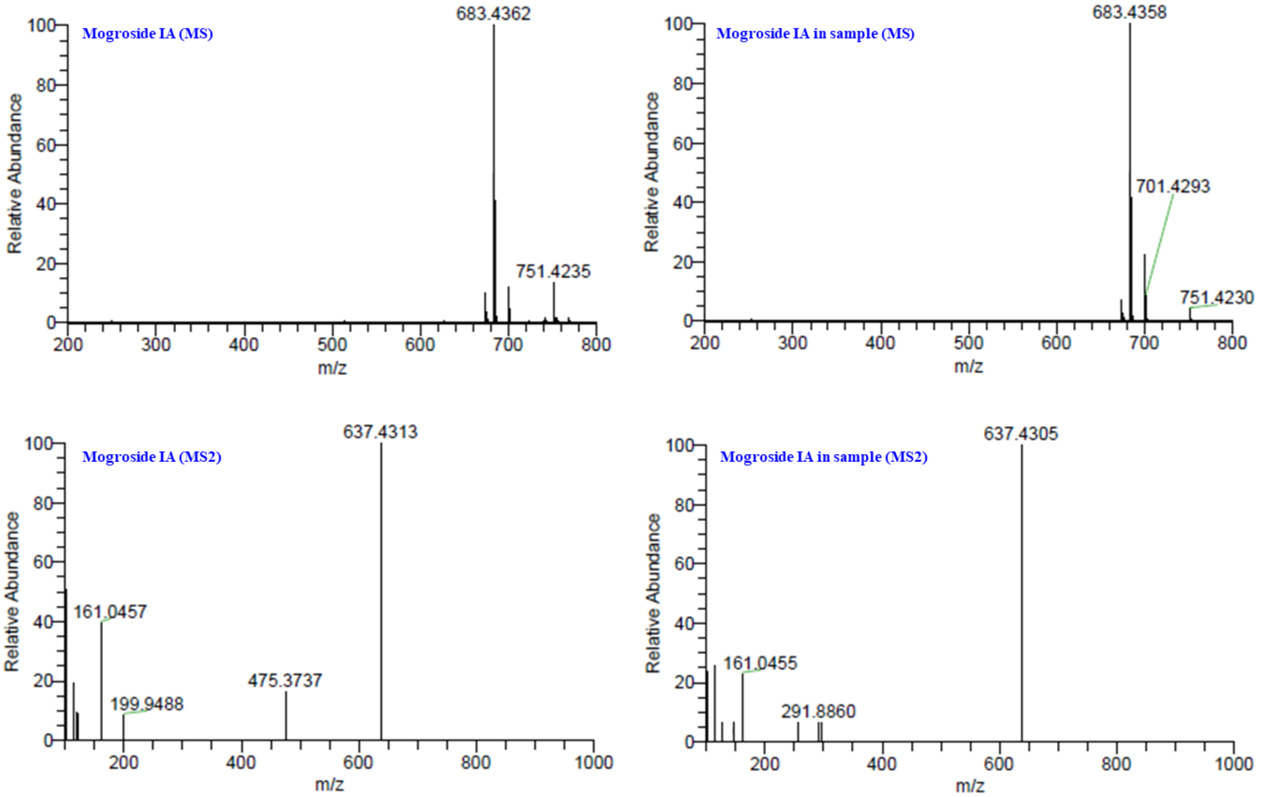

Supplementary Figure S1. The mass spectra in full-scan mode and targeted-MS2 mode for mogrosides with different glucose units. (a) The MS and MS2 of mogroside VI; (b) The MS and MS2 of mogroside V; (c) The MS and MS2 of siamenoside I; (d) The MS and MS2 of mogroside III; (e) The MS and MS2 of mogroside IIE; (f) The MS and MS2 of mogroside IA

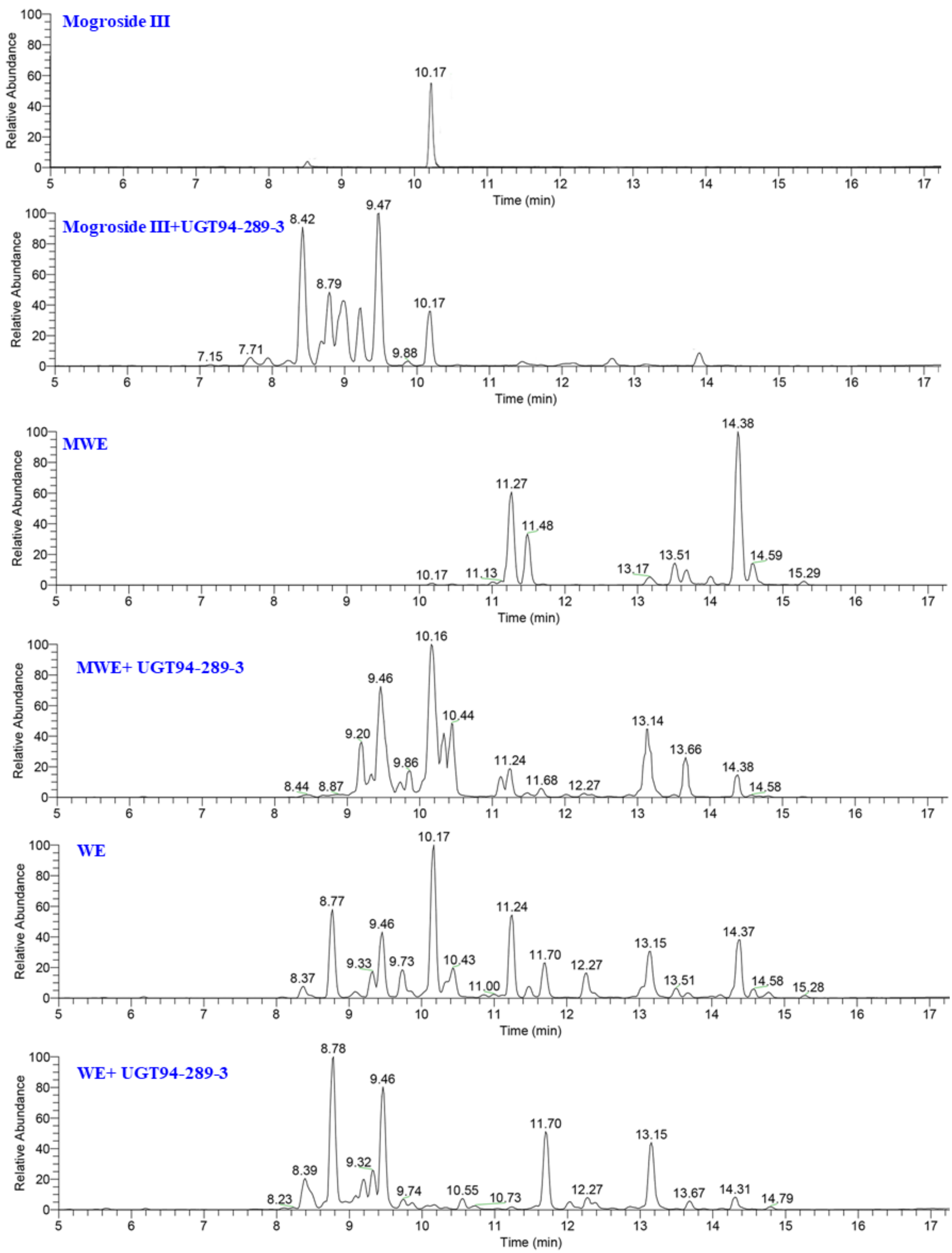

Supplementary Figure S2. Extraction ion chromatography of the catalytic products of mogroside III, MWE and WE.

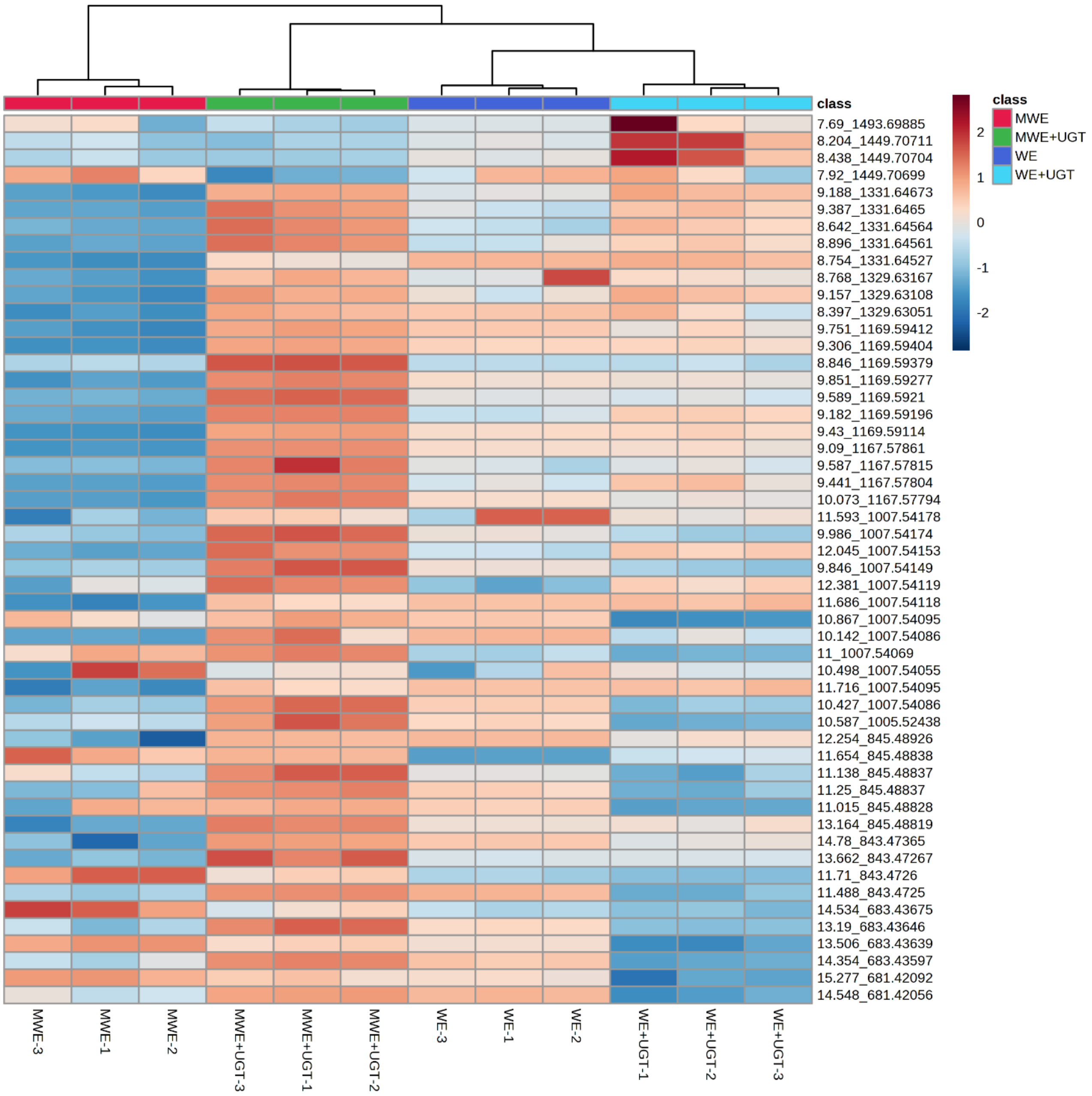

Supplementary Figure S3. Cluster heatmap of mogroside metabolites in the extracts and them catalytic products.

Supplementary Table S1. The results of the quantitative method validation.

mogrosides	**Linearity**		**LOQ**	**LOD**	**Precision (RSD, %)**		**Recovery% (RSD, %)**			**Repeatability**
	**Range (μg/mL)**	r^2	**(μg/mL)**	**(μg/mL)**	**Intra-Day**	**Inter-Day**	**0.5 ug/mL**	**2.0 ug/mL**	**5.0 ug/mL**	**(RSD, %)**
mogroside Ⅵ	0.5–20	0.9933	0.5	0.2	5.36	3.65	101.25 (5.82)	89.66 (6.21)	87.42 4.32)	5.46
mogroside Ⅴ	0.5–20	1	0.5	0.2	5.04	5.84	94.55 (6.24)	98.40 (6.09)	96.32 (6.55)	5.28
siamenoside Ⅰ	0.5–20	0.9995	0.5	0.2	8.76	4.21	99.86 (5.47)	93.45 (4.98)	96.41 (7.26)	6.41
mogroside ⅣA	0.5–20	0.9918	0.5	0.2	6.38	4.35	96.30 (5.06)	94.6 (6.554)	96.68(6.44)	8.05
mogroside Ⅳ	0.5–20	0.9987	0.5	0.2	6.77	5.5	96.00 (9.83)	99.65 (7.64)	97.40 (5.83)	4.68
mogroside Ⅲ	0.5–20	0.999	0.2	0.08	6.95	6.12	102.61 (3.54)	97.53 (4.87)	88.55(3.91)	3.74
mogroside ⅡE	0.5–20	0.9997	0.2	0.08	3.48	4.22	105.49 (4.78)	94.46(4.12)	98.53 (4.54)	3.56
mogroside IA	0.5–20	0.9998	0.02	0.01	2.74	7.01	98.65 (4.11)	100.58 (5.15)	97.57 (4.62)	5.91

Supplementary Table S2. The list of identified mogroside metabolites in the extracts and them catalytic products.

Compound ID	Compound Name	Retention Time (min)	Molecular Formula	Calc. MW	MS (m/z)	MS2 Fragment (m/z)
7.69_1493.69885	Isomer of mogroside VI	7.690	$C_{66}H_{112}O_{34}$	1448.7035	1447.6924;1493.7002	1447.6924;1285.6514;1123.5892;961.5258;799.4753;637.4352
8.204_1449.70711	Isomer of mogroside VI	8.204	$C_{66}H_{112}O_{34}$	1448.7035	1447.6924;1493.7002	1447.6924;1285.6514;1123.5892;961.5258;799.4753;637.4352
8.438_1449.70704	Isomer of mogroside VI	8.438	$C_{66}H_{112}O_{34}$	1448.7035	1447.6924;1493.7002	1447.6924;1285.6514;1123.5892;961.5258;799.4753;637.4352
7.92_1449.70699	Isomer of mogroside VI	7.920	$C_{66}H_{112}O_{34}$	1448.7035	1447.6924;1493.7002	1447.6924;1285.6514;1123.5892;961.5258;799.4753;637.4352
9.188_1331.64673	Isomer of mogroside Ⅴ	9.180	$C_{60}H_{102}O_{29}$	1286.6506	1331.6470;1285.6514	1285.6514;1123.5892;961.5258;799.4753;637.4352

9.387_1331.6465	Isomer of mogroside Ⅴ	9.387	$C_{60}H_{102}O_{29}$	1286.6506	1331.6470;1285.6514	1285.6514;1123.5892;961.5258;799.4753;637.4352
8.642_1331.64564	Isomer of mogroside Ⅴ	8.642	$C_{60}H_{102}O_{29}$	1286.6506	1331.6470;1285.6514	1285.6514;1123.5892;961.5258;799.4753;637.4352
8.896_1331.64561	Isomer of mogroside Ⅴ	8.896	$C_{60}H_{102}O_{29}$	1286.6506	1331.6470;1285.6514	1285.6514;1123.5892;961.5258;799.4753;637.4352
8.754_1331.64527	Mogroside Ⅴ	8.754	$C_{60}H_{102}O_{29}$	1286.6506	1331.6470;1285.6514	1285.6514;1123.5892;961.5258;799.4753;637.4352
8.768_1329.63167	Isomer of 11-Oxomogroside Ⅴ	8.768	$C_{60}H_{100}O_{29}$	1284.635	1329.6327;1283.6267	1283.6267;1121.5709;959.5239;797.4661;635.4319
9.157_1329.63108	Isomer of 11-Oxomogroside Ⅴ	9.157	$C_{60}H_{100}O_{29}$	1284.635	1329.6327;1283.6267	1283.6267;1121.5709;959.5239;797.4661;635.4319
8.397_1329.63051	11-Oxomogroside Ⅴ	8.397	$C_{60}H_{100}O_{29}$	1284.635	1329.6327;1283.6267	1283.6267;1121.5709;959.5239;797.4661;635.4319
9.751_1169.59412	Mogroside Ⅳ	9.751	$C_{54}H_{92}O_{24}$	1124.5978	1169.5940;1123.5892	1123.5892;961.5366;799.4835;637.4275
9.306_1169.59404	Siamenoside Ⅰ	9.306	$C_{54}H_{92}O_{24}$	1124.5978	1169.5940;1123.5892	1123.5892;961.5366;799.4835;637.4275

8.846_1169.59379	Isomer of Ⅳ	8.846	$C_{54}H_{92}O_{24}$	1124.5978	1169.5940;1123.5892	1123.5892;961.5366;799.4835;637.4275
9.851_1169.59277	Isomer of Ⅳ	9.851	$C_{54}H_{92}O_{24}$	1124.5978	1169.5940;1123.5892	1123.5892;961.5366;799.4835;637.4275
9.589_1169.5921	Isomer of Ⅳ	9.589	$C_{54}H_{92}O_{24}$	1124.5978	1169.5940;1123.5892	1123.5892;961.5366;799.4835;637.4275
9.182_1169.59196	Isomer of Ⅳ	9.182	$C_{54}H_{92}O_{24}$	1124.5978	1169.5940;1123.5892	1123.5892;961.5366;799.4835;637.4275
9.43_1169.59114	Mogroside ⅣA	9.430	$C_{54}H_{92}O_{24}$	1124.5978	1169.5940;1123.5892	1123.5892;961.5366;799.4835;637.4275
9.09_1167.57861	11-O-Siamenoside Ⅰ	9.090	$C_{54}H_{90}O_{24}$	1122.5822	1167.5787;1121.5725	1121.5725;959.5148;797.3799;635.4319
9.587_1167.57815	Isomer of 11-O-siamenoside Ⅰ	9.587	$C_{54}H_{90}O_{24}$	1122.5822	1167.5787;1121.5725	1121.5725;959.5148;797.3799;635.4319
9.441_1167.57804	Isomer of 11-O-siamenoside Ⅰ	9.441	$C_{54}H_{90}O_{24}$	1122.5822	1167.5787;1121.5725	1121.5725;959.5148;797.3799;635.4319
10.073_1167.57794	Isomer of 11-O-siamenoside Ⅰ	10.073	$C_{54}H_{90}O_{24}$	1122.5822	1167.5787;1121.5725	1121.5725;959.5148;797.3799;635.4319
11.593_1007.54178	Isomer of mogroside III	11.593	$C_{48}H_{82}O_{19}$	962.545	1007.5423;961.5368	961.5368;799.4839;637.4294;475.3731
9.986_1007.54174	Isomer of mogroside III	9.986	$C_{48}H_{82}O_{19}$	962.545	1007.5423;961.5368	961.5368;799.4839;637.4294;475.3731
12.045_1007.54153	Isomer of mogroside III	12.045	$C_{48}H_{82}O_{19}$	962.545	1007.5423;961.5368	961.5368;799.4839;637.4294;475.3731
9.846_1007.54149	Isomer of mogroside III	9.846	$C_{48}H_{82}O_{19}$	962.545	1007.5423;961.5368	961.5368;799.4839;637.4294;475.3731
12.381_1007.54119	Isomer of mogroside III	12.381	$C_{48}H_{82}O_{19}$	962.545	1007.5423;961.5368	961.5368;799.4839;637.4294;475.3731
11.686_1007.54118	Isomer of mogroside III	11.686	$C_{48}H_{82}O_{19}$	962.545	1007.5423;961.5368	961.5368;799.4839;637.4294;475.3731
10.867_1007.54095	Isomer of mogroside III	10.867	$C_{48}H_{82}O_{19}$	962.545	1007.5423;961.5368	961.5368;799.4839;637.4294;475.3731
10.142_1007.54086	Mogroside Ⅲ	10.142	$C_{48}H_{82}O_{19}$	962.545	1007.5423;961.5368	961.5368;799.4839;637.4294;475.3731

11_1007.54069	Isomer of mogroside III	11.000	$C_{48}H_{82}O_{19}$	962.545	1007.5423;961.5368	961.5368;799.4839;637.4294;475.3731
10.498_1007.54055	Isomer of mogroside III	10.498	$C_{48}H_{82}O_{19}$	962.545	1007.5423;961.5368	961.5368;799.4839;637.4294;475.3731
11.716_1007.54095	Mogroside ⅢA1	11.716	$C_{48}H_{82}O_{19}$	962.545	1007.5423;961.5368	961.5368;799.4839;637.4294;475.3731
10.427_1007.54086	Mogroside ⅢA2	10.427	$C_{48}H_{82}O_{19}$	962.545	1007.5423;961.5368	961.5368;799.4839;637.4294;475.3731
10.587_1005.52438	Isomer of 11-oxo-mogroside III	10.587	$C_{48}H_{80}O_{19}$	960.5294	1005.5281;959.5336	959.5336;797.4736;635.4147;161.0452
12.254_845.48926	Isomer of mogroside ⅡE	12.254	$C_{42}H_{72}O_{14}$	800.4922	845.4894;799.4837	799.4837;637.4323;161.0454
11.654_845.48838	Isomer of mogroside ⅡE	11.654	$C_{42}H_{72}O_{14}$	800.4922	845.4894;799.4837	799.4837;637.4323;161.0454
11.138_845.48837	Isomer of mogroside ⅡE	11.138	$C_{42}H_{72}O_{14}$	800.4922	845.4894;799.4837	799.4837;637.4323;161.0454
11.25_845.48837	Mogroside ⅡE	11.250	$C_{42}H_{72}O_{14}$	800.4922	845.4894;799.4837	799.4837;637.4323;161.0454
11.015_845.48828	Isomer of mogroside ⅡE	11.015	$C_{42}H_{72}O_{14}$	800.4922	845.4894;799.4837	799.4837;637.4323;161.0454
13.164_845.48819	Mogroside ⅡA1	13.164	$C_{42}H_{72}O_{14}$	800.4922	845.4894;799.4837	799.4837;637.4323;161.0454
14.78_843.47365	Isomer of 11-oxo-mogroside Ⅱe	14.780	$C_{42}H_{70}O_{14}$	798.4765	843.4745;797.4689	797.4689;635.4147+B1:G30;161.0454
13.662_843.47267	Isomer of 11-oxo-mogroside Ⅱe	13.662	$C_{42}H_{70}O_{14}$	798.4765	843.4745;797.4689	797.4689;635.4147+B1:G30;161.0454
11.71_843.4726	Isomer of 11-oxo-mogroside Ⅱe	11.710	$C_{42}H_{70}O_{14}$	798.4765	843.4745;797.4689	797.4689;635.4147+B1:G30;161.0454
11.488_843.4725	11-oxo-mogroside ⅡE	11.488	$C_{42}H_{70}O_{14}$	798.4765	843.4745;797.4689	797.4689;635.4147+B1:G30;161.0454
14.534_683.43675	Isomer of mogroside ⅠA	14.534	$C_{36}H_{62}O_{9}$	638.4393	683.4369;637.4324;475.3768	637.4324;475.3768;161.0454
13.19_683.43646	Isomer of mogroside ⅠA	13.190	$C_{36}H_{62}O_{9}$	638.4393	683.4369;637.4324;475.3768	637.4324;475.3768;161.0454
13.506_683.43639	Mogroside ⅠE	13.506	$C_{36}H_{62}O_{9}$	638.4393	683.4369;637.4324;475.3768	637.4324;475.3768;161.0454

14.354_683.43597	Mogroside ⅠA	14.354	$C_{36}H_{62}O_9$	638.4393	683.4369;637.4324;475.3768	637.4324;475.3768;161.0454
15.277_681.42092	Isomer of 11-oxo-mogroside ⅠA	15.277	$C_{36}H_{60}O_9$	636.4237	681.4211;635.4173	635.4173;161.0465
14.548_681.42056	Isomer of 11-oxo-mogroside ⅠA	14.548	$C_{36}H_{60}O_9$	636.4237	681.4211;635.4173	635.4173;161.0465

Supplementary Table S3. The quantitative results of eight mogrosides in extracts and its catalytic products.

Sample group	Content of mogrosides (mg/g)							
	mogroside Ⅵ	mogroside Ⅴ	siamenoside Ⅰ	mogroside ⅣA	mogroside Ⅳ	mogroside Ⅲ	mogroside ⅡE	mogroside IA
MWE	ND	ND	ND	ND	ND	1.34±0.27	37.35±4.57	18.82±1.29
MWE+UGT94-289-3	ND	ND	7.05±0.49	128.14±11.82	5.44±0.99	45.34±3.17	4.56±1.02	0.86±0.09
WE	ND	14.05±0.6	1.61±0.15	11.03±1.51	1.99±0.15	4.08±0.22	1.56±0.11	0.38±0.02
WE+UGT94-289-3	ND	16.34±2.48	1.45±0.35	17.56±3.07	0.53±0.06	0.17±0.03	ND	ND

Note: ND means not detected or below than LOQ.
